# Bortezomib Inpatient Prescribing Practices in Free-Standing Children's Hospitals in the United States

**DOI:** 10.1371/journal.pone.0151362

**Published:** 2016-03-15

**Authors:** Amanda M. DiNofia, Elizabeth Salazar, Alix E. Seif, Yimei Li, Yuan-Shung Vera Huang, Rochelle Bagatell, Brian T. Fisher, Richard Aplenc

**Affiliations:** 1 Division of Oncology, The Children’s Hospital of Philadelphia, Philadelphia, Pennsylvania, United States of America; 2 Department of Pediatrics, University of Pennsylvania Perelman School of Medicine, Philadelphia, Pennsylvania, United States of America; 3 Center for Clinical Epidemiology and Biostatistics, University of Pennsylvania Perelman School of Medicine, Philadelphia, Pennsylvania, United States of America; 4 Center for Pediatric Clinical Effectiveness, The Children’s Hospital of Philadelphia, Philadelphia, Pennsylvania, United States of America; 5 Division of Infectious Diseases, The Children’s Hospital of Philadelphia, Philadelphia, Pennsylvania, United States of America; Nottingham University, UNITED KINGDOM

## Abstract

This study is a pharmacoepidemiologic description of pediatric bortezomib use. Exposure was identified through billing codes in patients admitted to US children’s hospitals that participated with the Pediatric Health Information System between 2004 and 2013. Associated information on underlying diseases, demographics, institutional use, mortality, and physician type was collected. Exposure to bortezomib was identified in 314 patients. Hematologist/Oncologists prescribed half of the bortezomib used. Use increased during the study period. Inpatient volume was positively correlated with bortezomib utilization. Bortezomib use in pediatrics is increasing for a variety of diseases. Variation in use exists across institutions. Further studies are needed to characterize bortezomib’s efficacy in pediatric diseases.

## Introduction

Bortezomib is a selective, reversible inhibitor of the 26S proteasome, which is a key component of cell growth and apoptosis [1]. The US Food and Drug Administration (FDA) approved bortezomib in 2003 to treat multiple myeloma. In practice, bortezomib is used to treat both malignant and benign disorders [1]. Although lacking an approved pediatric indication, bortezomib is currently being tested to treat several malignancies [2–8], to prevent and reverse rejection of solid organ transplants [9–12], and to manage autoimmune cytopenias following bone marrow transplant (BMT) [13, 14]. In addition, bortezomib is used in refractory thrombotic thrombocytopenic purpura (TTP) [15]. However, the data supporting bortezomib use in children remains limited. There are small phase I and II pediatric studies of bortezomib in oncology that describe a total of 157 patients [2–8, 13]. The literature supporting bortezomib use in solid organ transplant and TTP are comprised of case reports and retrospective reviews [9–12, 15]. Despite the limited published data, bortezomib use in multiple pediatric subspecialties appears to be increasing across a diverse set of diseases.

Since little is known about the pharmacoepidemiology of bortezomib in children in the United States, we performed a descriptive analysis of bortezomib use in free-standing pediatric hospitals in the United States using the Pediatric Health Information System (PHIS) database. We hypothesized that bortezomib use would be most common in malignancies and that its use would increase over time in all patients. Postulating that patients receiving an off-label medication may have more advanced diseases, we also sought to describe the association between mortality and bortezomib administration. We were able to address these goals with our data.

## Methods

PHIS is an administrative database containing billing data from 46 tertiary children’s hospitals affiliated with the Children’s Hospital Association, which accounts for 85% of admissions to US freestanding children’s hospitals. PHIS data are composed of inpatient information including patient demographics, International Classification of Diseases, Ninth Revision, Clinical Modification (ICD-9-CM) diagnosis codes, and daily billing data for specific resources. PHIS data quality is overseen by the Children’s Hospital Association, Truven Health Analytics, and participating hospitals. This data is de-identified. The Children’s Hospital of Philadelphia Institutional Review Board does not consider this work to be human subjects’ research, and this project was granted exemption status.

The source population for the study included all patients who were admitted to PHIS-contributing hospitals between January 1, 2004 and December 31, 2013. Patients who received bortezomib during inpatient admissions were identified through pharmacy billing codes. Patients were followed for 30 days after the initial bortezomib exposure or the entirety of the last hospitalization that fell within 30 days from the initial exposure.

ICD-9 diagnosis codes reported during the first bortezomib admission were used to identify the underlying diseases of patients included in the cohort. A previously described method was used to group patients by underlying disease type [16]. The following categories were used: leukemia; lymphoma and solid tumor malignancies; solid organ and bone marrow transplant; and “other” diseases, which included diagnosis codes that did not fall into any of the former diagnostic groups. Patients with an ICD-9 diagnosis code that mapped to more than one category were classified according to the predefined order: leukemia, lymphoma and solid tumors, or transplant. In addition to the diagnoses used to categorize the underlying primary disease of patients exposed to bortezomib, each hospital identified a principle diagnosis that they deemed is the main reason for the hospital admission. We tabulated the most common principle diagnoses attributed to patients exposed to bortezomib.

Patient demographics, total inpatient days, mortality, and bortezomib use by institution were summarized by standard descriptive statistics for the overall population and for each disease category. In-hospital mortality was estimated with 95% CIs. The treating physician during the first bortezomib admission was designated as the prescriber. We calculated total bortezomib use by institution, controlling for years each institution contributed data to PHIS, and compared that number to the average inpatient volume at that institution.

## Results

A total of 314 patients from the 46 PHIS institutions were identified with a billing code for bortezomib during the study period. Table 1 illustrates the characteristics of the study population at the first admission in which bortezomib was billed. Bortezomib use was greatest in the leukemia and transplant disease categories. Diagnoses in the “other” category included autoimmune cytopenias and complications of heart and kidney disease. Although these patients with heart and kidney diseases did not have a transplant diagnosis code mapping them to the transplant disease category, their bortezomib exposure likely reflects that they did receive a solid organ transplant, since bortezomib use is only described in these populations in the anti-rejection setting. Bortezomib use was evenly distributed between ages 1 and 22 years in the leukemia and transplant categories; however, older children were treated with bortezomib more than younger children in the lymphoma and solid tumors category. When evaluating all patients treated with bortezomib, regardless of disease category, 56% were less than 15 years of age. Fifty-eight percent of the bortezomib-exposed patients were male, and 64% were white. Nearly half were publically insured.

**Table 1:** Characteristics of Study Population.

		Underlying Disease Category			
	All patients (n = 314)	Leukemia (n = 119)	Lymphoma and solid malignancies (n = 54)	Transplant (n = 119)	Other (n = 22)
Age group, Freq. (Percent)					
< 1 yr.	14 (5)	5 (4)	0	5 (4)	4 (18)
≥ 1 and < 5 yrs.	54 (17)	19 (16)	5 (9)	24 (20)	6 (27)
≥ 5 and < 10 yrs.	49 (16)	25 (21)	5 (9)	15 (13)	4 (18)
≥ 10 and <15 yrs.	60 (19)	26 (22)	10 (19)	21 (18)	3 (14)
≥ 15 and <18 yrs.	59 (19)	16 (14)	18 (33)	24 (20)	1 (5)
≥ 18 and <22 yrs.	61 (19)	20 (17)	12 (22)	28 (24)	1 (5)
≥ 22 yrs	17 (5)	8 (7)	4 (7)	2 (2)	3 (14)
Sex, Freq. (Percent)					
Male	181 (58)	66 (56)	23 (43)	77 (65)	15 (68)
Race, Freq. (Percent)					
Caucasian	201 (64)	76 (64)	34 (63)	75 (63)	16 (73)
Insurance, Freq. (Percent)					
Private	136 (43)	56 (47)	29 (54)	42 (35)	9 (41)
Public^a^	148 (47)	58 (49)	17 (32)	66 (56)	7 (32)
Other^b^	30 (10)	5 (4)	8 (15)	11 (9)	6 (27)

^a^ Public includes Medicaid, Medicare, Other government, and Title V.

^b^ Other includes Self-pay, Other, and Unknown.

The majority (85%) of the patients had only one bortezomib admission within 30 days from the initial bortezomib exposure with only 13% having two bortezomib admissions and 1% having three admissions. The range of total inpatient days varied in each category, but the medians clustered around 30 inpatient days. When evaluating the frequency of bortezomib use by pediatric physician specialty, pediatric hematology/oncology comprised 50% of bortezomib use, consistent with the finding that 55% of patients receiving bortezomib had either leukemia or lymphoma/solid tumor diagnoses, followed by cardiology (17%) and nephrology (9%). The ten most common principle diagnoses associated with a hospitalization that included a bortezomib exposure are listed in Table 2. These diagnoses accounted for 214 patients or 68% of the cohort.

**Table 2:** Principle Diagnoses Associated with Hospitalization that Included Bortezomib Exposure.

Diagnosis	Number of Patients
Encounter for antineoplastic chemotherapy	66
Complications of transplanted heart	38
Complications of transplanted kidney	32
Acute lymphoid leukemia, in relapse	24
Acute myeloid leukemia	15
Complications of transplanted lung	12
Congestive heart failure	9
Acute lymphoid leukemia	7
Complications of transplanted liver	6
Hypoplastic left heart syndrome	5

**Fig 1:**
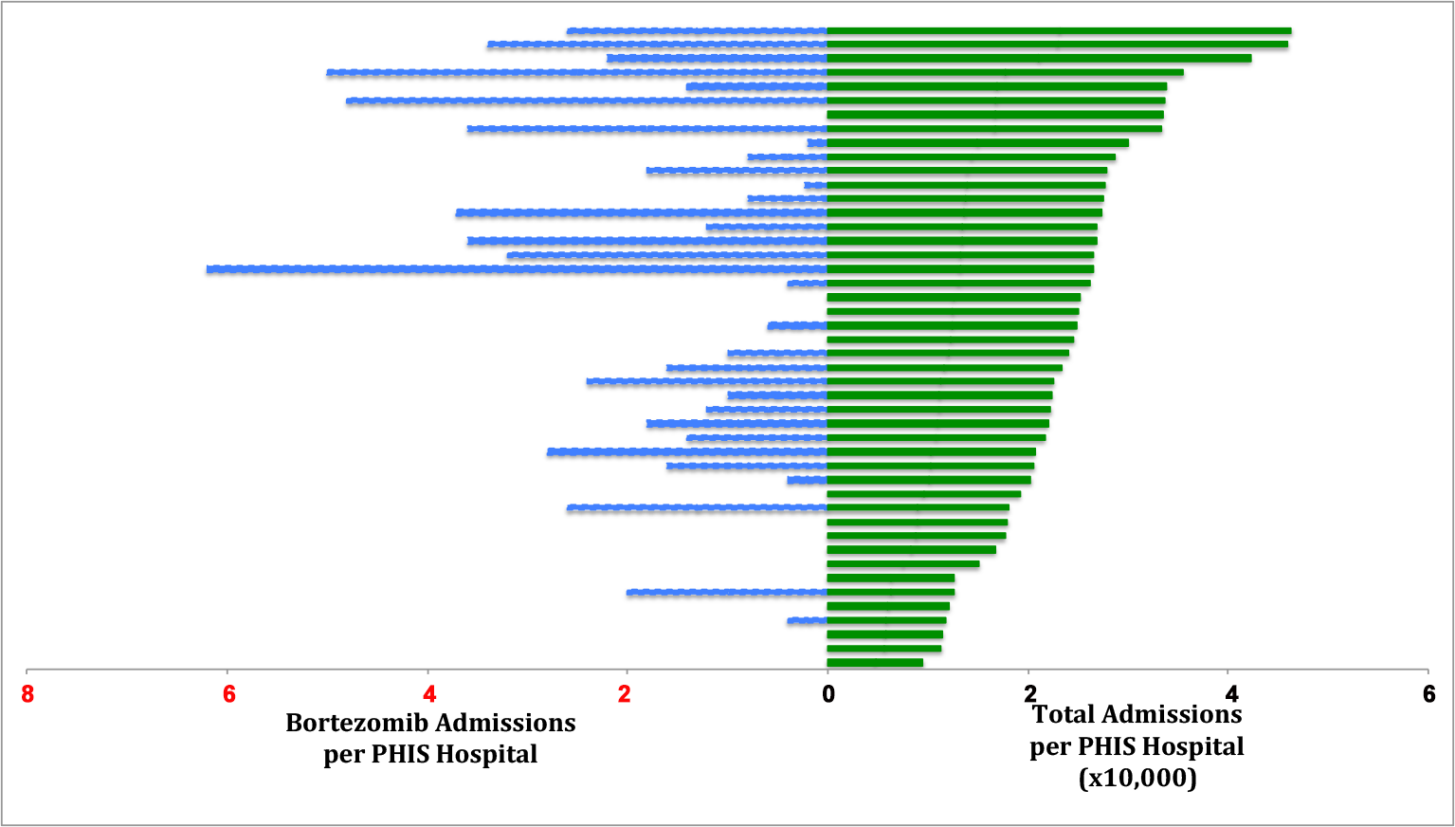
Bortezomib Admissions per Hospital vs Total Admissions per Hospital (x10,000) from 2004–2013. Legend: Blue line–bortezomib admissions per PHIS hospital, Green line–total admissions per PHIS hospital (x10,000).

Bortezomib use increased over the data collection period from 2 patients with bortezomib billing data out of 38 PHIS hospitals in 2004 to 85 patients out of 46 hospitals in 2013. Fig 1 depicts the number of bortezomib admissions per year in each institution plotted against the total admissions per year in that institution over the study period. Hospitals with lower total admission rates had less bortezomib utilization (ρ 0.50, p = 0.004). The predominant prescribers at two of the three hospitals with the greatest utilization of bortezomib were hematologists and oncologists. Nephrologists were the leading prescribers at the other high utilizing institution.

The in-hospital mortality for patients exposed to bortezomib within the study window was 12% (37 of 314, 95%CI 8.4%-15.9%). The disease category with the highest percentage of mortality was the transplant category (13%, 16 of 119, 95%CI 7.3%-19.6%), and the lowest frequency was in the lymphoma and solid malignancy category (7%, 4 of 54, 95% CI 2.1%-17.9%).

## Discussion

The 314 patients identified in our study represent the largest series of pediatric patients exposed to bortezomib in the literature and provide the first pharmacoepidemiologic description of bortezomib in the pediatric population. The current literature and our data suggest that most of pediatric bortezomib use outside of oncology is to prevent graft rejection after solid organ transplant [9–11]. Despite the absence of FDA approval, bortezomib use in pediatrics is steadily increasing. There is clearly variation in utilization among institutions that is associated with total hospital volume. Larger hospitals may have more medically complex patients that fail standard therapies or may have more experience with experimental therapies that contributes to a lower threshold for off-label medication use.

Almost half of the patients treated with bortezomib were greater than 15 years of age. This high proportion of exposed adolescents likely reflects increased physician comfort with treating patients closer in age to adults for whom bortezomib use is FDA-approved. More male children were treated with bortezomib than females. It is unclear if this discrepancy is due to a difference in proportion of standard therapy failure between males and females or a gender disparity, which has been demonstrated in renal transplantation access [17]. The proportion of bortezomib use between white and non-white children reflects the distribution of race in the United States [18].

The mortality rate in hospitalizations that included bortezomib administration was 12%. This high inpatient mortality likely reflects the seriousness of the underlying illnesses for which bortezomib is currently being used. While this increase in mortality rate could be directly related to bortezomib exposure, the descriptive nature of this study precludes the determination of causality between bortezomib and mortality.

The inability to establish a causal relationship between inpatient mortality and bortezomib exposure without a comparator group is an important limitation of this study. In addition, conclusions on bortezomib effectiveness and toxicity are not possible with this dataset. An additional limitation is that bortezomib utilization is based on pharmacy billing codes. Since bortezomib is a newer drug, some institutions may not have a billing code for bortezomib, which would result in an underestimation of the frequency of bortezomib usage. Finally, many patients in the “other” category had diagnoses reflecting heart and kidney complications. These patients likely received solid organ transplants despite not having a transplant diagnosis code. There is likely an underestimation of exposed patients in the transplant category.

Despite these limitations, our study demonstrates that bortezomib is being used across pediatric subspecialties for various indications, and there is practice variation among hospitals. The center-level characteristics that drive hospitals to be early adopters of new drugs without FDA approval should be further explored. Delays in pediatric specific pharmaceutical investigations, while rooted in ensuring drug safety, have resulted in children being exposed to experimental agents in the absence of data. Future research should also focus on conducting randomized controlled and comparative effectiveness trials to better comprehend bortezomib’s value in different disease processes and describe its risk profile in pediatrics.
